# A Tale of Two Disasters

**DOI:** 10.1093/geroni/igaa057.2429

**Published:** 2020-12-16

**Authors:** Tammy Mermelstein

**Affiliations:** University of Houston - Downtown, Houston, Texas, United States

## Abstract

Preparing for or experiencing a disaster is never easy, but how leaders communicate with older adults can ease a situation or make it exponentially worse. This case study describes two disasters in the same city: Hurricane Harvey and the 2018 Houston Texas Ice Storm and the variation in messaging provided to and regarding older adults. For example, during Hurricane Harvey, the primary pre-disaster message was self-preparedness. During the storm, messages were also about individual survival. Statements such as “do not [climb into your attic] unless you have an ax or means to break through,” generated additional fear for older adults and loved ones. Yet, when an ice storm paralyzed Houston a few months later, public messaging had a strong “check on your elderly neighbors” component. This talk will explore how messaging for these events impacted older adults through traditional and social media analysis, and describe how social media platforms assisted people with rescue and recovery. Part of a symposium sponsored by Disasters and Older Adults Interest Group.

